# EvoSeg: Automated Electron Microscopy Segmentation through Random Forests and Evolutionary Optimization

**DOI:** 10.3390/biomimetics6020037

**Published:** 2021-06-01

**Authors:** Manuel Zumbado-Corrales, Juan Esquivel-Rodríguez

**Affiliations:** Pattern Recognition and Machine Learning Group, Computer Engineering School, Costa Rica Institute of Technology, Cartago 30101, Costa Rica; manzumbado@ic-itcr.ac.cr

**Keywords:** electron microscopy, segmentation, EM, machine learning, random forests, evolutionary algorithms

## Abstract

Electron Microscopy Maps are key in the study of bio-molecular structures, ranging from borderline atomic level to the sub-cellular range. These maps describe the envelopes that cover possibly a very large number of proteins that form molecular machines within the cell. Within those envelopes, we are interested to find what regions correspond to specific proteins so that we can understand how they function, and design drugs that can enhance or suppress a process that they are involved in, along with other experimental purposes. A classic approach by which we can begin the exploration of map regions is to apply a segmentation algorithm. This yields a mask where each voxel in 3D space is assigned an identifier that maps it to a segment; an ideal segmentation would map each segment to one protein unit, which is rarely the case. In this work, we present a method that uses bio-inspired optimization, through an Evolutionary-Optimized Segmentation algorithm, to iteratively improve upon baseline segments obtained from a classical approach, called watershed segmentation. The cost function used by the evolutionary optimization is based on an ideal segmentation classifier trained as part of this development, which uses basic structural information available to scientists, such as the number of expected units, volume and topology. We show that a basic initial segmentation with the additional information allows our evolutionary method to find better segmentation results, compared to the baseline generated by the watershed.

## 1. Introduction

The biological sciences have greatly benefited from advances in microscopy over the past decade, which have enabled techniques such as cryo-electron microscopy to produce 3D images that elucidate near-atomic level features of proteins. The highest resolution images created have approached the 1.5 Å range [1,2,3]. At this resolution, it is even possible to identify atomic structures in proteins, although the results depend on the map itself. Identifying protein structures at lower resolutions is still a crucial task to understand the way macromolecular assemblies work, as the average resolution of structures released in public databases remains over 5 Å [4]. Several methods have been developed to identify the secondary structure of proteins within EM maps [5,6,7,8,9]. A related problem, de novo chain modeling, has been addressed by leveraging segmentation and symmetry [10]. More recently, the use of machine learning techniques has been applied in this space [11,12,13,14] and deep learning, in particular, has shown its potential to identify secondary structure elements in EM maps [15,16,17,18]. It is possible to use hybrid approaches to extract additional information from electron microscopy maps at resolutions up to 10 Å [19]. Similar techniques have been applied to study the organization of proteomes, as seen in [20]. Furthermore, the same algorithms have been applied to the EM-fitting problem domain, where we want to identify how an atomic resolution protein model fits into an EM map, with the objective of knowing the exact position of each element [21,22,23].

The atomic level details found in a map are not the only valuable pieces of information. In [24] it was possible to find the architecture and helical regions of a 26S proteasome. It is also possible to use the information encoded in EM maps to study the interactions between previously unknown protein complexes [25].

The development of algorithms and models in this domain has been boosted by the large availability of open bio-molecular data. On one hand, the Protein Data Bank (PDB) [26] is a well known resource that contains over 175,000 atomic-resolution protein, RNA and DNA structural complexes. While some of the structures deposited in the PDB correspond to larger molecules, the majority of them only contain a handful of protein units.

To complement this high-resolution data source, we can also leverage a second valuable source of data: the EM Data Resource [27]. This database stores three-dimensional models derived from electron microscopy data that allows us to study larger bio-molecular complexes, although at lower resolutions than we would normally find with PDB data. Most notably, the majority of EM maps do not easily allow for atomic identification. Another important characteristic of these maps is that, due to the lower resolution of images found, we need to deal with the noise that comes in the images. According to the repository statistics, while the average resolution of EM structures deposited went from roughly 12 Å in 2015 to about 6 Å in recent years, the mean resolution across all maps is still over 5 Å. At such resolutions, the identification of atomic elements is still a challenge, which requires computational methods that help elucidate structural insights.

A common algorithmic technique employed to study the possible molecular units contained in an EM map is based on image segmentation principles. The main idea is to divide maps into density regions that should correspond to individual protein units or, at minimum, relevant domains within protein units. Some of the approaches assume prior knowledge about the components, or the symmetry between protein complexes [28,29,30]. The atomic-detailed models that correspond to EM maps under study may not always be available or we may need to deal with additional complexities, in order to obtain baseline EM maps that yield lower resolution images [31].

The immersive watershed algorithm is a classic technique used to assign density regions to labels, as a first pass, followed by a grouping stage where we can use methods such as scale-space filtering [32]. As explained by the authors, that method is useful to avoid over-segmenting images. A prior study applying this type of segmentation in EM maps was shown in [33]. At a high level, the watershed algorithm can be understood as a process by which we can fill topographical regions with water, as an analogy, by first allowing the entry through local minima that can represent small holes [34]. As we submerge the regions further down into the water, the level will rise from the minima entry points progressively. So called watershed lines are defined whenever two distinct regions are about to touch through the water, as the level rises. All these lines would delineate the different segments.

Even when such approaches can be better than basic segmentation, it is still common to find that EM images are over segmented, as we have seen in [33]. It can be possible to use classical segmentation implementations such as *Segger* [32] but the burden on the scientists interested in analyzing molecular structures can be high, as they need to fine tune parameters that can vary from one case to another. Ideally, we want computational methods that only require basic information about the molecules under study that can be used to find segmentation candidates that improve upon the baseline given by watershed segmentation, for instance. It is not unreasonable to think that scientists already have access to the approximate volume of individual protein units, the approximate number of units expected and some information about the connectivity between units. We can then use the additional information to iteratively search for better segmentation results.

In the following sections we describe a methodology that combines these concepts within the framework of evolutionary algorithms. This type of optimization technique has already been proven successful to optimize watershed segmentation in multi-spectral images [35]. The overall effectiveness of such algorithms has also been shown in the general field of medical imaging [35,36,37].

The main contribution of our work centers around improved segmentation results against state-of-the-art segmentation, such as *Segger* [32]. In order to achieve this we ask for a small amount of information that describes the molecule, such as the expected volume and connectivity between units. The additional data given allow us to differentiate between good and bad segmentation candidates, as our method iteratively optimizes using the evolutionary framework.

As we will see in our results, both from a quantitative and a qualitative point of view, we are able to improve upon baseline segmentation candidates without requiring extensive parameter tuning from researchers.

## 2. EvoSeg: Evolutionary-Optimized Segmentation

The ideal segmentation can be defined as one where individual regions in maps match one corresponding protein unit of the macromolecular assembly. The flip side of this would be to have protein units that contain multiple segments or even segments that span across multiple proteins.

We frame the problem to solve as a two-stage investigation where the end result should be to find a close-to-ideal segmentation. In order to do so, we require:

- A way of determining if a segmentation is good or bad. In other words, is a segmentation result close to being biologically correct?
- Once we can discriminate good segmentation results, we need a method to create segmentation candidates to explore the space, and keep traversing the regions of the space that are biologically relevant.

**Figure 1:**
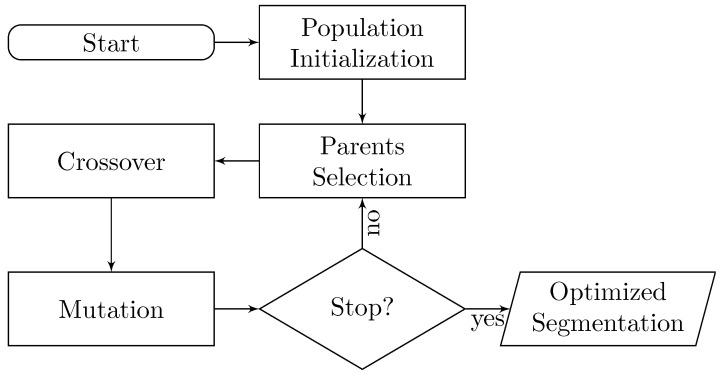
Diagram of the Evolutionary-Optimized Segmentation protocol proposed. The optimization process relies on a supervised classifier trained on a curated dataset of relevant EM maps. Parameters and feature encoding are defined in detail, in Section 2.

For the first part, we created a supervised learning classifier, trained on a dataset suited for this purpose, described in Section 2.4. Thanks to the existence of the classifier, we can perform an evolutionary optimization process over a set of segmentation states. This stochastic process flow is shown in Figure 1. First, we define an initial population as a set of random segmentation states. Successive states are generated combining two parent states, as explained in Section 2.1. Each created state is subjected to random mutations, a process detailed in Section 2.2. Then, the fitness value of each individual is determined by how close it is to a meaningful solution of the problem (i.e., the ground truth segmentation). A Random Forest classifier is trained to learn the fitness function from EM and PDB data. Details about fitness scoring and the data representation used to learn the fitness function are presented in Section 2.3 and Section 2.4, respectively. Finally, the iterative process stops when the proposed fitness goals are achieved.

In general, a state (or “individual”) with a high fitness score equates to having a higher probability of being selected as parent to create new individuals; this process follows a round-robin approach. The number of individuals in the initial population is set to 30, mostly due to resource constraints when running the algorithm. There is a σ parameter that controls the segmentation smoothing amount in each region grouping step *s*, as defined by the scale-space grouping process. σ and *s* values follow a uniform distribution between 1 and 4. A fitness value is assigned to each individual after the initial population is created. Then, 30 parents are selected according to the round robin policy and combined to create 15 new segmentation states. Individuals with the best fitness score have a higher chance of being parents multiple times. With respect to the stopping criteria, the optimization process stops either at 200 iterations or after 20 consecutive iterations without reaching a higher fitness score. As we will see in Section 4, there is convergence within those boundaries.

### 2.1. Crossover

As introduced in Figure 1, crossover is performed after parents are selected, specifically with a probability of 0.9. Otherwise, the offspring is just a direct copy of one of its parents, uniformly selected. If we define the set of *n* selected parents as $\Delta =\{\delta_{1},\delta_{2},\ldots ,\delta_{n}\}$ in a single iteration, each offspring created by the combination of two different parents is the result of the crossover operator defined as follows:

(1) $$X\;=\;\delta_{a}\;⊗\;\delta_{b},$$

with X a set of *r* segments $\left\{\chi_{1},\chi_{2},\ldots ,\chi_{r}\right\}$ where $X\;\subset \;\delta_{a}\;\cup \;\delta_{b}$. Note that a parent is also a set of segments. Thus, the set of segments of an offspring is determined by the uniform selection and superposition of segments from its parents, iteratively. The combination stops when there are no voxels left without an associated semantic class label.

### 2.2. Mutation

Mutation is performed on offspring with a probability of 0.1. We define two mutation operations based on the most common observations around segmentation errors. Over segmentation is the most recurring issue we have seen, where segmentation algorithms conservatively create small sections without merging areas that, in the context of bio-molecules, are part of just one unit, but the density profile is different enough that the algorithms do not consider them to be “mergeable”. Similarly, for regions with fuzzy boundaries, where density profiles are too similar, there is a risk that the initial segmentation joins areas that are meant to be separate.

#### 2.2.1. Merge Operation

This operation addresses the first error case described. A single segment *S* in the offspring segmentation individual is randomly selected. Adjacent segments are considered candidates to be merged with the segment selected *S*. A new segment is created with a new semantic class label by the union of voxels of *S* with voxels of one of its adjacent segments, uniformly selected.

#### 2.2.2. Split Operation

A split is in charge of repairing segmentation problems due to fuzzy boundaries in density space. This is performed on a single segment *Q* of the offspring individual. A new random watershed segmentation $Q^{\prime }$ is computed, so that *Q* is split in two or more regions of $Q^{\prime }$ that match the voxels of *Q*. In some cases, voxels of segment *Q* are associated to only one segment in $Q^{\prime }$. In that case, a new random segmentation is computed until splitting becomes feasible.

### 2.3. Ideal Segmentation Classifier

Our method uses a feature representation of map segments for the purpose of segmentation evaluation. The ideal segmentation is the perfect matching of predicted segments to their corresponding protein units, in the ground truth. In other words, an ideal segmentation is obtained when the predicted segments have the same structural details of ground truth proteins.

Selected features encode structural details of a segmented map, in particular:

- Number of segments: Directly derived from the expected number of protein units in the macromolecule.
- Volume of segment: Proportion of the total macromolecule volume in voxels, captured by each segment.
- Topology of segments: Euler–Poincaré Characteristic is used to encode invariant shape details of segments.

Following a supervised training approach, the learning algorithm has to produce a function $f:R^{n}\to R^{k}$ where *k* is the number of classes. When y=f(x) the model assigns an input described by the vector of features x to a probability distribution over *k* classes denoted as y. In our case, the model has to be able to distinguish between a bad and a good segmentation. That is, the model outputs a probability value for a segmentation to be classified as bad or good. The value of interest here is the probability that ends up becoming the fitness function that describes individuals in the Evolutionary-Optimized Segmentation process.

As mentioned above, the feature vector x encodes structural information of a segmented map. In detail, $x=(x_{1},x_{2},\ldots ,x_{n})$ where n=121. The first component of the feature vector x encodes the number of identified segments, overall. The remaining components encode 2 elements per segment: the volume and its correspondent Euler–Poincaré characteristic (key due to its shape invariance). Based on the distribution of segments in the ground truth, we define an upper bound to the maximum number of segments to encode at 60 (for a total of 120 components that describe individual segments). We set component values to zero when the macromolecule under study does not require all (i.e., because it has less than the maximum segments to be encoded).

In order to get the best model parameters, a grid search is performed over parameter search spaces and models as presented in Table 1. Because our focus is not just on creating a classifier, but to use it as a way of identifying good segmentation candidates, we use a vanilla grid search approach, where standard parameter values for each of the models is used. The order of magnitude of the numeric parameters is similar, for each of the parameters mentioned, and we report the parameter values that yielded the highest area under the ROC curve for each model. We select Random Forests since it yields the best results.

### 2.4. Data

**Figure 2:**
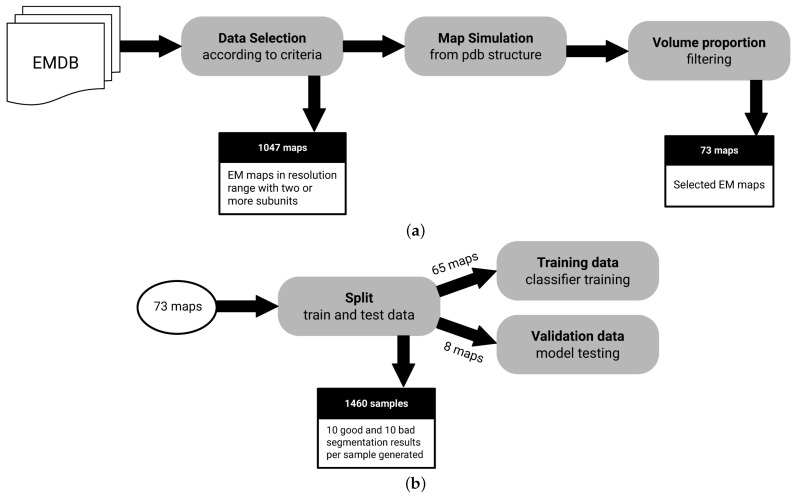
Dataset creation process. (**a**) All EMDB entries are analyzed to filter candidates according to criteria described in Section 2.4, resulting in 73 maps available. (**b**) Dataset is split in standard training and test sets, as well as an 8 map holdout set. Note that samples are augmented to create bad and good segmentation cases, since the size of our data pool could be a concern in training.

To the best of our knowledge, there is no fully-annotated EM segmentation dataset available for the evaluation of a method, such as the one proposed here. We require electron microscopy maps such that there is an annotated segment identifier that links each voxel, in map space, to an actual protein unit. To create such dataset, we use the EM Data Resource (EMDB) and the Protein Data Bank (PDB) public databases. EM maps with a matching atomic structure on the Protein Data Bank allow us to create fully-annotated cases. Figure 2 shows a diagram that summarizes the dataset creation process.

In total, 12,667 EM maps are automatically analyzed to filter candidate maps according to the following selection criteria:

- Macromolecules with at least 2 subunits: We need macromolecules with more than a single subunit, as we want to assess segmentation performance.
- Macromolecules in a resolution range of 4.5 Å to 10 Å: It is possible to derive structures from maps with resolutions below 4.5 Å. On the other hand, lower resolutions above 10 Å may not have enough signal for predictive purposes.
- Macromolecules with an associated atomic structure: It is necessary for ground truth annotation.

After the selected samples are filtered using the criteria described above, an additional volume proportion filter is used to exclude maps where the volume contained in the map is not proportional to the volume generated by their associated atomic structure. This is an ad hoc check that we had to add at some point for high-throughput matching of PDBs, since some structures that allegedly were related in metadata did not directly match as would be expected. The atomic structure has the information needed to associate regions of maps to an specific molecular subunit, allowing the creation of ground truth. The selected proportional volume ratio between the map and the atomic structure has to be a value between 0.8 and 1.2.

The filtering process results in a dataset of 73 maps. To train the classification model, a data augmentation approach is used to create a balanced number of samples annotated accordingly to bad and good segmentation classes. Bad segmentation samples are generated with watershed which typically produces over segmentation. Good segmentation samples are derived from ground truth annotation of segments, randomly selecting and splitting a single segment per map. In the end, 20 samples (10 annotated as good and 10 as bad) are augmented from each sample of the filtered dataset, resulting in 1460 samples.

## 3. Performance Measures

### 3.1. Matching IoU

Because the segmentation we produce can have a different number of segments than the ground truth, a challenge we are faced with is how to correctly reward and penalize particular cases. As an example, if the ground truth had a map with two segments $\Gamma =\{\gamma_{1},\gamma_{2}\}$ and we have a candidate with three segments $P=\{\rho_{1},\rho_{2},\rho_{3}\}$ we would want to penalize more heavily if all $\rho_{i}$ span between both ground truth segments frequently. However, we may not want to penalize as significantly if $\gamma_{1}\approx \rho_{1}$ and $\gamma_{2}\approx \rho_{2}$, because $\rho_{3}$ could be a small volume which does not affect the overall segmentation result. It is good to remember that our objective in this study is to provide a meaningful segmentation to scientists studying protein complexes, which may not be significantly impacted by small segmentation discrepancies.

**Figure 3:**
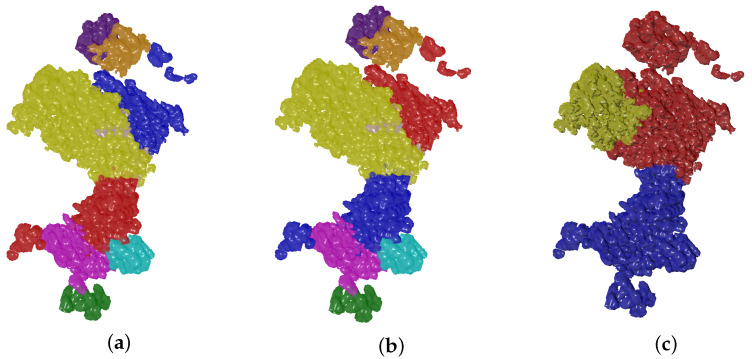
Selected segmentation results are evaluated on ground truth data using a matched version of the IoU metric. In this Figure, regions of a selected segmentation result of EMD-0044 (**a**) are permuted (**b**) to match ground truth labels (**c**).

IoU is one of the standard metrics to evaluate segmentation tasks. We follow a similar approach as the one presented in [38] to address the problem of label permutation of segmentation results to match ground truth, and its invariance in qualitative validation. Let the ground truth labeling of a map denoted by Γ to have *t* segments $\left\{\gamma_{1},\gamma_{2},\ldots ,\gamma_{t}\right\}$ where each segment is a macromolecule subunit given by a set of voxels with an associated semantic class label. The output of the Evolutionary-Optimized Segmentation is a set P of *p* segments $\left\{\rho_{1},\rho_{2},\ldots ,\rho_{p}\right\}$, each associated with a semantic class label too. As mentioned before, *t* and *p* values could vary such that a segmentation result can lead to less, equal or more segments than ground truth value. We can denote Φ as a set with all possible label permutations on regions of the resulting segmentation P. Note that in Figure 3 permutations between red and blue region labels of the selected segmentation result do not have an effect in the qualitative result. Thus, $\overset{˚}{P}$ can be defined as the matched permutation that maximizes the IoU between the segmentation result P and the ground truth Γ:

(2) $$\overset{˚}{P}=\underset{\phi \in \Phi }{\mathrm{arg max}}\;\mathrm{IoU}\left(\phi ,\Gamma \right)\;.$$

### 3.2. Homogeneity

Every segment should be as homogeneous as possible. In other words, when our algorithm creates a segment, ideally, it should only contain voxels that correspond to a single segment from the ground truth. More specifically, we want to measure how many voxels in a segment $\gamma_{i}$ correspond to the correct ground truth segment $\rho_{j}$ (i.e., true positives). However, because perfect segments are unlikely to happen, we want to know how many of the voxels in $\gamma_{i}$ correspond to some other ground truth segment $\rho_{k}$ (i.e., false positives). We thus repurpose the canonical definition of precision to be our homogeneity measure, which we would want to be as high as possible.

(3) $$H=\frac{\mathrm{TP}}{\mathrm{TP}+\mathrm{FP}}.$$

Note that we can have *H* values with an over segmentation. In particular, if every ground truth $\rho_{j}$ has multiple predicted segments $\gamma_{i}$ we can maximize the metric by having every segment be a single voxel assign to the correct $\rho_{j}$. We call this out in order to make it explicit that *H* alone would not be enough to evaluate a good segmentation.

### 3.3. Proportion of Estimated Segments

In order to address the cases not captured by *H* we also want to measure, for each ground truth segment, the number of predicted segments $s_{i}$ that were created. The ideal case, naturally, is that one estimated segment would correspond to one ground truth segment (i.e., a protein unit). The larger this proportion is, the further away it would be from a good segmentation. Because this metric should take into account all segments, we normalize it by the actual number of segments *N*.

(4) $$P=\frac{1}{N}\sum_{i=1}^{N}s_{i}.$$

### 3.4. Consistency

An average can dilute the extreme effect that point values have. Since *P* is an average we also want to understand what the most dominant assignment is, for particular segments. We analyze the protein unit (i.e., a ground truth segment) with the largest number of predicted segments to serve as a consistency measure. If the largest protein has a diverse number of predicted segments, then the overall prediction is not likely to be useful.

(5) $$C=P_{\max(i)}.$$

## 4. Results

The main objective at the onset of this work was to create a computational protocol that would allow scientists (e.g., biologists, pharmacologists, physicians) to start from an Electron Microscopy map, gather some basic information about the proteins contained in the EM and produce better segmentation results than what baseline segmentation can produce at this time. In this section, we show that we have achieved that goal by highlighting the metrics described previously, as well as comparing against segmentation results obtained in ChimeraX [39], a well known bio-molecular manipulation tool with segmentation features.

### 4.1. Ideal Segmentation Classifier

The main evolutionary optimization protocol is in charge of generating the final segmentation results. Before analyzing them, however, we first highlight the results of training a Random Forest classifier that serves as the cost function in the optimization process.

In Table 1 we show the Area Under the ROC curve for several model alternatives we tried, with the best parameters tested. To do this we used the dataset described in Section 2.4 using the standard cross-validation procedure with a holdout validation set.

**Figure 4:**
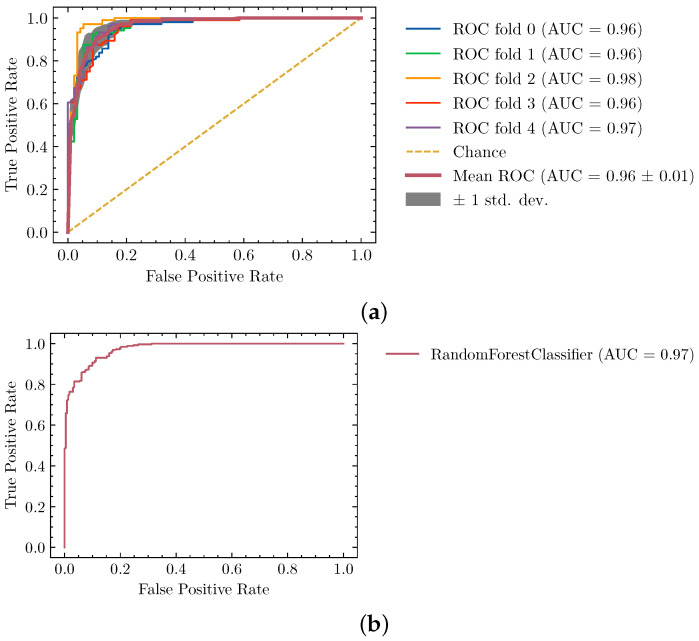
Performance of the segmentation classifier described by (**a**) training and (**b**) validation ROC curves. Generalization power is evaluated with a final testing dataset of 8 maps previously unseen by the model.

We provide more detailed results about the Random Forest training and validation process in Figure 4, where we can see that the training was stable across folds and the validation set, along the 2 axes of the ROC curve. Note that because the classifier is a key component to the overall protocol we decided to use the majority of our available dataset in the cross-validation process. That is the reason why only eight unseen structures are used for final validation.

It is worth pointing out that using supervised learning here requires us to have a complete set of EM maps and matching ground truth PDB structures. That is a key limiting factor in terms of the size of the dataset available. While there are other less rigorous avenues to create a synthetic dataset (e.g., generating predictions on EM maps using software tools) we preferred to use experimentally validated data in the form of deposited Protein Data Bank structures.

### 4.2. Evolutionary-Optimized Segmentation Validation

As mentioned in Section 4.1, we have used a large number of structures from the dataset in the cross validation process, to train the ideal segmentation classifier. If we were to assess the effectiveness of the evolutionary optimization using any of the elements in that portion of the dataset we could be giving us an unfair advantage, and thus make the results appear better than they truly are.

For that reason, in this section we show detailed analysis of eight EM structures, which were not seen by the classifier training process. We have calculated all four metrics described in Section 3, which can be seen in Table 2. We provide their fitness score along with the other measures.

The range of both **Matching IoU** and **H** is between $\left[0,1\right]$ while **C** and **P** are >=1. In the case of the former two, we want higher values; the latter ones should have as low a value as possible. To provide a fair interpretation for the metrics we need to establish that a perfect segmentation with limited information is very difficult to achieve. Furthermore, as we have pointed out in previous sections, EM maps are inherently noisy. We can categorize five out of the eight results as successful, in terms of providing a segmentation that has a good coverage based on **Matching IoU** and **H**, and provides a low number of predicted segments per ground truth protein unit.

We complement this analysis by comparing our method against *Segger*, which can be considered the state-of-the-art segmentation, and it is included in some of the most recognized structural biology tools. In Table 2, we also present *Segger* IoU results. As we can see, our method is able to obtain better results in all cases. Note that both IoU metrics calculated in the table are the most strict assessment we can apply to both methods, since there can be a series of segments that do not have a correspondence to a ground truth segment. This is to be expected since over segmentation is a common issue, in this type of problem. That means that the (potentially) large number of zero IoU values will bring the metric down, when aggregated overall.

To determine whether the unmatched segments are affecting either of the methods, we also calculated IoU taking into account only segments that were matched to a ground truth counterpart. Table 3 shows that comparison. Our method outperforms *Segger* in four of the cases, it has comparable results in two, and it is outperformed in the remaining two cases (EMD-0044 and EMD-3573).

We also compare the number of segments generated by each of the two methods in Table 4. The main reason to do this is to understand the possible over segmentation yielded. We can see that we reduce the number of predicted segments by an order of magnitude in every case. We are not able to match the ground truth number of segments but, given our objective, this is clearly an improvement over *Segger*. Note how there is a large discrepancy in the number of segments generated for the 2 cases where our method is outperformed by *Segger* in Table 3.

**Figure 5:**
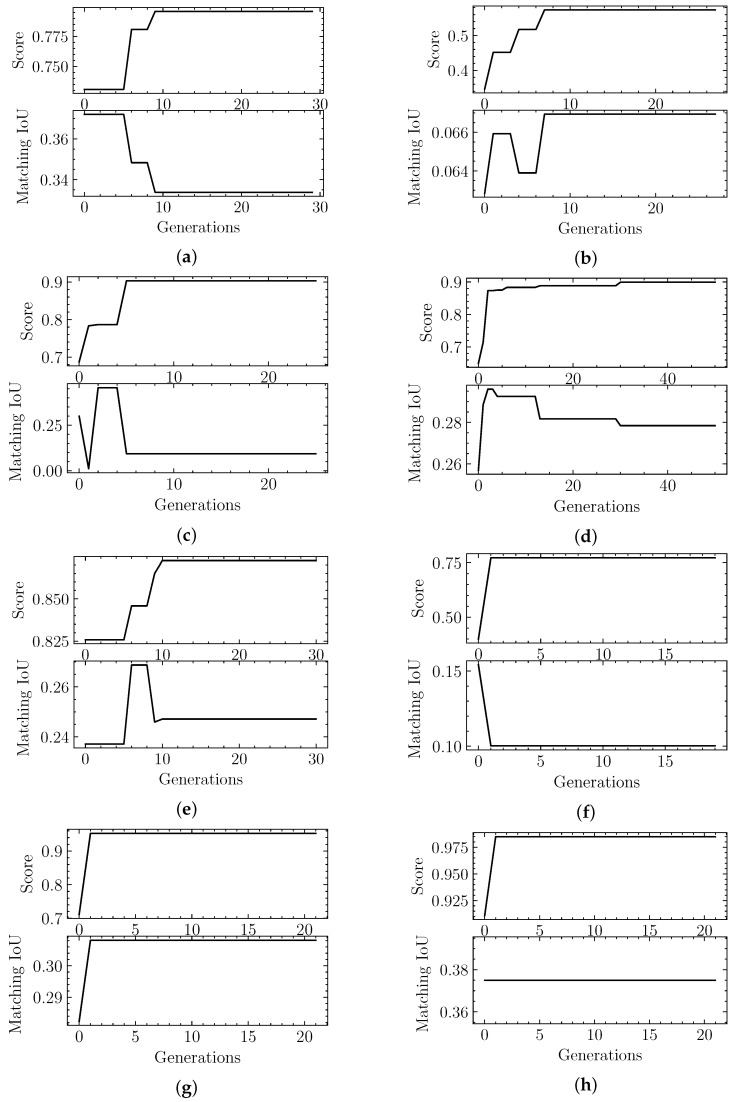
Score and Matching IoU metric evolution through successive generations by our Evolutionary-Optimized Segmentation applied to testing data: (**a**) EMD-7090, (**b**) EMD-4670, (**c**) EMD-3573, (**d**) EMD-0044, (**e**) EMD-0790, (**f**) EMD-8528, (**g**) EMD-3206, (**h**) EMD-20824. Note that in (**a**), (**c**)–(**f**) even if a higher fitness score is achieved throughout the generations, the Matching IoU is not always the highest attained.

When we analyzed the results in Table 2 we stated that three of the eight validation cases should be considered worse results, based on the metrics. We looked into the evolutionary optimization logs, shown in Figure 5, to determine whether it was a search problem (i.e., the algorithm never explored a good region of the space) or a fitness function problem (i.e., the classifier evaluates bad segmentation candidates as good), in order to inform future improvements.

We find that, in the case of EMD-3573, there was a better candidate structure earlier in the exploration process, but the fitness score did not deem it to be as good as other segmentation predictions. This case points to a problem directly related to the fitness function, which could be solved with the exposure of the classifier to more data. Given that our dataset was limited to start with, we may need to use data augmentation techniques or use some portion of synthetic data. Either potential approach has pros and cons that would need to be explored in a future study.

The other two cases, EMD-4670 and EMD-8528, were never able to find a good candidate in terms of the fitness score, which points to a search problem. As stated in Section 2 we already have stopping criteria in place that ensure that the candidates are stable for a large number of generations. In Section 5 we mention some alternatives that can be pursued in the future to address this problem.

It is important to note that all runs shown in Figure 5 allow all of the population to be replaced from one generation to the next. That is why it can sometimes yield significant fluctuations. Table 5 showcases the summary results using a strategy that maintains the top fitness scores from one generation to the next, shown as “Result 2” (“Result 1” corresponds to Figure 5 results). We can see that, from an optimization point of view, the convergence at the end is similar. We must note, however, that the actual IoU results, which is our true target, finds better results in some cases.

**Figure 6:**
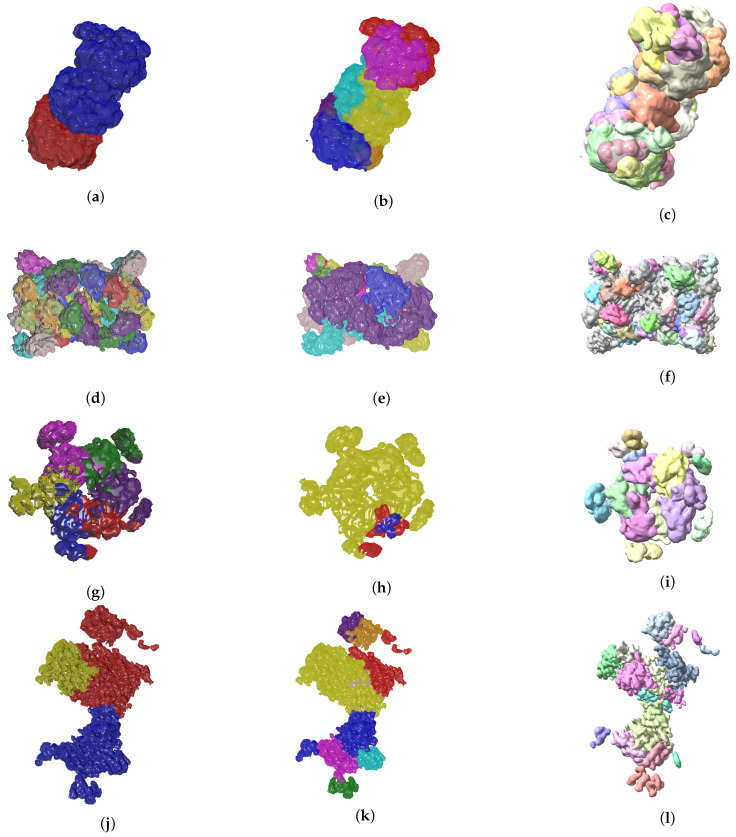
Ground truth labels of testing data, the Evolutionary-Optimized Segmentation and *Segger* segmentation results are shown on the left, center and right columns, respectively. The listed maps are EMD-7090 ((**a**)–(**c**)), EMD-4670 ((**d**)–(**f**)), EMD-3573 ((**g**)–(**i**)), EMD-0044 ((**j**)–(**l**)). Results show that baseline *Segger* produces segmentation results with a number of segments far from the ground truth ideal value, in contrast to our method.

**Figure 7:**
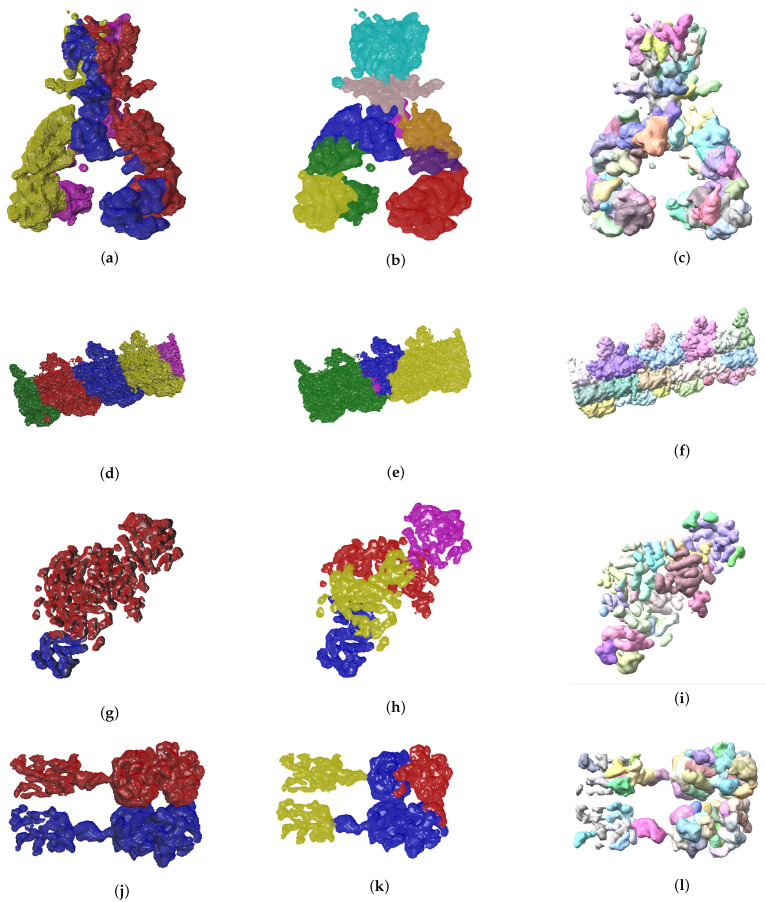
Ground truth labels of testing data, EvoSeg and *Segger* segmentation results are shown on the left, center and right columns respectively. The listed maps are EMD-7090 ((**a**)–(**c**)), EMD-0790 ((**d**)–(**f**)), EMD-3206 ((**g**)–(**i**)), EMD-20824 ((**j**)–(**l**)).

So far we have presented measures to highlight the effectiveness of our method. In the context of structural biology, it is always good to visualize the results because they should be consistent with metrics gathered. In Figure 6 and Figure 7 we see three visual representations for each of the 8 validation cases:

- The ground truth protein units
- Our segmentation results
- *Segger* results with baseline configuration

We can see that we can consistently provide segmentation results that have less segments and are closer to the target structures, especially for the cases where our metrics show good results. While it could be possible to configure *Segger* so that a better segmentation result is generated, the parameterization can be very specific to each case, as opposed to the basic information our method requires.

## 5. Conclusions and Discussion

In this work we have shown that it is possible to obtain more refined segmentation results with respect to baseline watershed segmentation, by using easily derivable information from protein complexes and an Evolutionary-Optimized segmentation algorithm, that is powered by a Random Forest classifier to assess good segmentation candidates. Existing tools in structural biology, such as ChimeraX [39], provide access to well established algorithms for segmentation but default configurations lead to over segmented images, in most cases. Better segmentation results can be achieved by manually optimizing parameters for each EM map under analysis. Our underlying hypothesis was that requiring information like basic topology and approximate volumes would allow us to provide improved results, without the need to fine tune difficult to understand segmentation configuration.

The results shown in Figure 6 and Figure 7 are clear from a qualitative standpoint. Specifically, we can see how the output generated by our method is closer to the ground truth segmentation for most of the cases. From the quantitative analysis point of view we see good metrics. Given the complexity of the problem at hand we consider Matching IoU values between 0.3 and 0.4 very promising particularly because the most common problem we are faced with is over segmentation, which can be solved with a marginal amount of human intervention. While it would be ideal to have a fully automated segmentation, and that is what we aim for, there is always domain knowledge that we can leverage from scientists who study these systems on a regular basis. As mentioned before, it is common for researchers to use computational tools to help in their work. Here we have made that tooling even closer to the ground truth. We recognize that, even though we have improved the segmentation over our baseline, there is still work to do in the future to push the boundaries of automated identification further.

For instance, there are several cases in which we see a better candidate segmentation throughout the evolutionary optimization, but we eventually replace it by a segmentation that is marginally worse. That can be interpreted as our search strategy being successful but leaving room for improvement on the cost function side. We can either augment our feature vector and eventually retrain our classifier or even expose the models to an augmented data set that allows it to learn even more segmentation patterns. We can also improve our population management by applying structural clustering to each population so that candidates kept from one generation to the next are as varied as possible. The idea in the context of protein complexes has previously been applied in [40], although adjustments would be needed, since we do not have atomic positions to guide the clustering, in the current problem domain.

All of the programmatic work we have developed and the data set used for training purposes is available in GitHub, as referenced in the Data Availability Statement.

## Figures and Tables

**Table 1:** Performance of different classification models with best parameters given by grid search. Random Forests outperforms other models in terms of the area under the ROC curve metric.

Model	Area under ROC	Parameters
Logistic Regression	0.77	C: 10, dual: False, max_iter: 120, penalty: l2
SVM	0.79	C: 0.1, gamma: 1, kernel: rbf
MLP	0.78	activation: relu, alpha: 0.0001,hidden_layer_sizes: 100, learning_rate: constant, solver: adam
Random Forests	0.83	max_features: auto, n_estimators: 1200

**Table 2:** Fitness score and segmentation evaluation metrics on validation dataset. Both IoU columns show the standard Intersection-Over-Union metric where our method outperforms *Segger* baseline segmentation (state-of-the-art comparison). *H* stands for Homogeneity, *C* for Consistency and *P* for Proportion. A value of *H* less than 1 means that subunits are partially captured by a smaller segment on average. *C* shows the number of segments identified in the subunit with largest volume. Column *P* describes the average number of different segments identified in a single subunit.

Sample	Score	Matching IoU	Segger IoU	H	C	P
EMD-7090	0.98	0.07	0.00	0.08	9	8.00
EMD-4670	0.57	0.08	0.06	0.25	1	3.35
EMD-0044	0.90	0.09	0.05	0.20	1	4.33
EMD-0790	0.87	0.11	0.00	0.14	5	5.00
EMD-8528	0.77	0.10	0.07	0.28	2	2.60
EMD-3206	0.95	0.15	0.02	0.35	4	2.50
EMD-3573	0.90	0.19	0.14	0.52	1	1.67
EMD-20824	0.98	0.23	0.00	0.40	3	3.00

**Table 3:** Matching IoU metric for EvoSeg and Segger. Matched segments were considered only, excluding remaining segments in the calculation of IoU metric.

Sample	Matching IoU	Segger IoU
EMD-7090	0.33	0.32
EMD-4670	0.07	0.07
EMD-0044	0.28	0.32
EMD-0790	0.25	0.13
EMD-8528	0.10	0.10
EMD-3206	0.31	0.26
EMD-3573	0.09	0.44
EMD-20824	0.37	0.08

**Table 4:** Number of segments for EvoSeg and Segger on the validation dataset. Results show that our method produces significantly less segments than baseline Segger.

Sample	EvoSeg	Segger	Ground Truth
EMD-7090	9	32	2
EMD-4670	44	293	54
EMD-0044	9	18	3
EMD-0790	9	91	4
EMD-8528	5	25	5
EMD-3206	4	31	2
EMD-3573	3	19	6
EMD-20824	3	81	2

**Table 5:** Matching IoU metric on matched segments and maximun score for two EvoSeg runs on the validation dataset.

Sample	Result 1 IoU	Result 1 Max Score	Result 2 IoU	Result 2 Max Score
EMD-7090	0.33	0.98	0.53	0.94
EMD-4670	0.07	0.57	0.08	0.55
EMD-0044	0.28	0.90	0.33	0.94
EMD-0790	0.25	0.87	0.29	0.96
EMD-8528	0.10	0.77	0.17	0.62
EMD-3206	0.31	0.95	0.38	0.98
EMD-3573	0.09	0.90	0.45	0.85
EMD-20824	0.37	0.98	0.37	0.98

## Data Availability

The code for all our computational biology initiatives is openly available at https://github.com/tecdatalab/biostructure (accessed on 1 April 2021). Data used for training purposes is available at https://github.com/tecdatalab/datasets/tree/main/evolutionary_segmentation (accessed on 1 April 2021). The main program to run EvoSeg, as well as a Jupyter Notebook, is provided at https://github.com/tecdatalab/biostructure/tree/master/em/evolutionary_segmentation (accessed on 1 April 2021). Note that, depending on the structure processed, more computational power may be needed. All our experiments were run using the infrastructure provided by the National Colaboratory for Advanced Computing at the National Center for High Technology (CNCA-CeNAT).

## Abbreviations

The following abbreviations are used in this manuscript:

EM	Electron Microscopy
PDB	Protein Data Bank
EvoSeg	Evolutionary-Optimized Segmentation

## References

[1] Zhang K., Pintilie G.D., Li S., Schmid M.F., Chiu W. (2020). Resolving Individual-Atom of Protein Complex using Commonly Available 300-kV Cryo-electron Microscopes. bioRxiv.

[2] Nakane T., Kotecha A., Sente A., McMullan G., Masiulis S., Brown P.M., Grigoras I.T., Malinauskaite L., Malinauskas T., Miehling J. (2020). Single-particle cryo-EM at atomic resolution. bioRxiv.

[3] Yip K.M., Fischer N., Paknia E., Chari A., Stark H. (2020). Atomic-resolution protein structure determination by cryo-EM. Nature.

[4] EMStats: EMDB Statistics. https://www.ebi.ac.uk/pdbe/emdb/statistics_sp_res.html/.

[5] Baker M.L., Baker M.R., Hryc C.F., Ju T., Chiu W. (2012). Gorgon and pathwalking: macromolecular modeling tools for subnanometer resolution density maps. Biopolymers.

[6] Baker M.L., Ju T., Chiu W. (2007). Identification of secondary structure elements in intermediate-resolution density maps. Structure.

[7] Jiang W., Baker M.L., Ludtke S.J., Chiu W. (2001). Bridging the information gap: computational tools for intermediate resolution structure interpretation. J. Mol. Biol..

[8] Kong Y., Ma J. (2003). A structural-informatics approach for mining beta-sheets: locating sheets in intermediate-resolution density maps. J. Mol. Biol..

[9] Kong Y., Zhang X., Baker T.S., Ma J. (2004). A Structural-informatics approach for tracing beta-sheets: building pseudo-C(alpha) traces for beta-strands in intermediate-resolution density maps. J. Mol. Biol..

[10] Terashi G., Kagaya Y., Kihara D. (2020). MAINMASTseg: Automated Map Segmentation Method for Cryo-EM Density Maps with Symmetry. J. Chem. Inf. Model..

[11] Chen L., Jebril R., Nasr K.A. (2020). Segmentation-Based Feature Extraction for Cryo-Electron Microscopy at Medium Resolution.

[12] Ng A., Si D. (2018). Beta-Barrel Detection for Medium Resolution Cryo-Electron Microscopy Density Maps Using Genetic Algorithms and Ray Tracing. J. Comput. Biol..

[13] Si D., Ji S., Nasr K.A., He J. (2012). A Machine Learning Approach for the Identification of Protein Secondary Structure Elements from Electron Cryo-Microscopy Density Maps. Biopolymers.

[14] Bajaj C., Goswami S., Zhang Q. (2012). Detection of secondary and supersecondary structures of proteins from cryo-electron microscopy. J. Struct. Biol..

[15] Subramaniya S.R.M.V., Terashi G., Kihara D. (2019). Protein secondary structure detection in intermediate-resolution cryo-EM maps using deep learning. Nat. Methods.

[16] He J., Huang S.Y. (2021). Full-length de novo protein structure determination from cryo-EM maps using deep learning. bioRxiv.

[17] Wang X., Alnabati E., Aderinwale T.W., Subramaniya S.R.M.V., Terashi G., Kihara D. (2020). Emap2sec+: Detecting Protein and DNA/RNA Structures in Cryo-EM Maps of Intermediate Resolution Using Deep Learning. bioRxiv.

[18] Mostosi P., Schindelin H., Kollmannsberger P., Thorn A. (2020). Haruspex: A Neural Network for the Automatic Identification of Oligonucleotides and Protein Secondary Structure in Cryo-Electron Microscopy Maps. Angew. Chem. Int. Ed..

[19] Lindert S., Stewart P.L., Meiler J. (2009). Hybrid approaches: applying computational methods in cryo-electron microscopy. Curr. Opin. Struct. Biol..

[20] Beck M., Topf M., Frazier Z., Tjong H., Xu M., Zhang S., Alber F. (2011). Exploring the spatial and temporal organization of a cell’s proteome. J. Struct. Biol..

[21] Dou H., Burrows D.W., Baker M.L., Ju T. (2017). Flexible Fitting of Atomic Models into Cryo-EM Density Maps Guided by Helix Correspondences. Biophys. J..

[22] Topf M., Baker M.L., John B., Chiu W., Sali A. (2005). Structural characterization of components of protein assemblies by comparative modeling and electron cryo-microscopy. J. Struct. Biol..

[23] Fabiola F., Chapman M.S. (2005). Fitting of High-Resolution Structures into Electron Microscopy Reconstruction Images. Structure.

[24] Beck F., Unverdorben P., Bohn S., Schweitzer A., Pfeifer G., Sakata E., Nickell S., Plitzko J.M., Villa E., Baumeister W. (2012). Near-atomic resolution structural model of the yeast 26S proteasome. Proc. Natl. Acad. Sci. USA.

[25] Hryc C.F., Chen D.H., Afonine P.V., Jakana J., Wang Z., Haase-Pettingell C., Jiang W., Adams P.D., King J.A., Schmid M.F. (2017). Accurate model annotation of a near-atomic resolution cryo-EM map. Proc. Natl. Acad. Sci. USA.

[26] Burley S.K., Berman H.M., Bhikadiya C., Bi C., Chen L., Costanzo L.D., Christie C., Duarte J.M., Dutta S., Feng Z. (2019). Protein Data Bank: The single global archive for 3D macromolecular structure data. Nucleic Acids Res..

[27] Lawson C.L., Patwardhan A., Baker M.L., Hryc C., Garcia E.S., Hudson B.P., Lagerstedt I., Ludtke S.J., Pintilie G., Sala R. (2016). EMDataBank unified data resource for 3DEM. Nucleic Acids Res..

[28] Baker M.L., Yu Z., Chiu W., Bajaj C. (2006). Automated segmentation of molecular subunits in electron cryomicroscopy density maps. J. Struct. Biol..

[29] Terwilliger T.C., Adams P.D., Afonine P.V., Sobolev O.V. (2018). A fully automatic method yielding initial models from high-resolution cryo-electron microscopy maps. Nat. Methods.

[30] Volkmann N. (2002). A novel three-dimensional variant of the watershed transform for segmentation of electron density maps. J. Struct. Biol..

[31] Patwardhan A., Brandt R., Butcher S.J., Collinson L., Gault D., Grünewald K., Hecksel C., Huiskonen J.T., Iudin A., Jones M.L. (2017). Building bridges between cellular and molecular structural biology. eLife.

[32] Pintilie G.D., Zhang J., Goddard T.D., Chiu W., Gossard D.C. (2010). Quantitative analysis of cryo-EM density map segmentation by watershed and scale-space filtering, and fitting of structures by alignment to regions. J. Struct. Biol..

[33] Manuel Z.C., Luis C.V., José S.B., Julio V.M., Daisuke K., Juan E.R. (2020). Matching of EM Map Segments to Structurally-Relevant Bio-molecular Regions.

[34] Vincent L., Soille P. (1991). Watersheds in digital spaces: an efficient algorithm based on immersion simulations. IEEE Trans. Pattern Anal. Mach. Intell..

[35] Derivaux S., Lefevre S., Wemmert C., Korczak J. On Machine Learning in Watershed Segmentation. Proceedings of the 2007 IEEE Workshop on Machine Learning for Signal Processing.

[36] Maulik U. (2009). Medical Image Segmentation Using Genetic Algorithms. IEEE Trans. Inf. Technol. Biomed..

[37] Javadpour A., Mohammadi A. (2016). Improving brain magnetic resonance image (MRI) segmentation via a novel algorithm based on genetic and regional growth. J. Biomed. Phys. Eng..

[38] Arnab A., Torr P.H.S. Pixelwise Instance Segmentation With a Dynamically Instantiated Network. Proceedings of the IEEE Conference on Computer Vision and Pattern Recognition (CVPR).

[39] Pettersen E.F., Goddard T.D., Huang C.C., Meng E.C., Couch G.S., Croll T.I., Morris J.H., Ferrin T.E. (2021). UCSF ChimeraX: Structure visualization for researchers, educators, and developers. Protein Sci..

[40] Esquivel-Rodríguez J., Yang Y.D., Kihara D. (2012). Multi-LZerD: Multiple protein docking for asymmetric complexes. Proteins.

